# Peripheral Humoral Immune Response Is Associated With the Non-motor Symptoms of Parkinson’s Disease

**DOI:** 10.3389/fnins.2019.01057

**Published:** 2019-10-10

**Authors:** Congcong Sun, Wenfei Yu, Zhenxiang Zhao, Chengyuan Song, Ying Liu, Guoyong Jia, Xingbang Wang, Yiming Liu

**Affiliations:** ^1^Department of Neurology, Qilu Hospital, Shandong University, Jinan, China; ^2^Department of Neurology, Henan Provincial People’s Hospital, People’s Hospital of Zhengzhou University, Zhengzhou, China

**Keywords:** non-motor symptoms, Parkinson’s disease, humoral immunity, serum complement, serum immunoglobulin

## Abstract

**Background:**

Non-motor symptoms are common in Parkinson’s disease (PD) and can even be used as part of the supportive criteria for diagnosis. Chronic inflammation is involved in every stage of PD. Disorders of the immune system affect the peripheral blood. Whether the humoral immune response is associated with the non-motor symptoms of PD remains unknown.

**Methods:**

Mann–Whitney tests and Bonferroni correction were used to compare the serum levels of IgG, IgA, IgM, C3, and C4 between 180 sporadic PD patients and 187 healthy controls. Multiple regression models were conducted to assess the associations among these indicators of humoral immunity and the clinical features of PD patients.

**Results:**

Male PD patients had lower levels of C3 and C4 than healthy controls [0.87 (0.22) vs. 0.96 (0.19); 0.19 (0.06) vs. 0.22 (0.07), respectively, Pc < 0.01] and lower levels of C3 than female PD patients [0.87 (0.22) vs. 1.02 (0.23), Pc < 0.01]. Patients suffering from attention/memory problems had significantly lower levels of IgA and C3 than those without these problems [1.92 (1.21) vs. 2.57 (0.76); 0.89 (0.24) vs. 0.97 (0.24), respectively, Pc < 0.04]. In addition, serum IgG levels were negatively associated with mood/cognition problem scores and were positively associated with gastrointestinal tract problem scores (adjusted *R*^2^ = 0.063, *F* = 1.805, *p* = 0.038). Serum C3 levels were negatively associated with being male, age, and sleep/fatigue problem scores (adjusted *R*^2^ = 0.123, *F* = 2.678, *p* = 0.001).

**Conclusion:**

The peripheral humoral immune response might be correlated with the non-motor symptoms of PD.

## Introduction

Parkinson’s disease (PD) is a common neurodegenerative disease characterized by alpha-synuclein (α-syn) deposition and a loss of dopaminergic neurons in the substantia nigra (SN) (Rocha et al., 2018). Apart from the cardinal motor manifestations, non-motor symptoms (NMSs) can also be seen in over 50% of PD patients (Durcan et al., 2019). Although the underlying mechanism remains elusive, neuroinflammation is considered to be involved in α-syn transmission and dopaminergic neuronal degeneration (Hirsch and Hunot, 2009; Butkovich et al., 2018).

Disorders of the immune system affect not only the central nervous system but also the humoral immune response in the peripheral blood (Boyko et al., 2017). Inflammatory cytokines, such as IL-1β, IL-2, IL-6, TNF-α, and IFN-γ, are altered in the sera of patients with PD (Gelders et al., 2018; Yilmaz et al., 2018). Several of these indicators were reported to be associated with NMSs (Lindqvist et al., 2012; Pereira et al., 2016; Kim et al., 2018). However, few studies have explored the role of immunoglobulins and the complement system in PD.

The immunoglobulins are important components of humoral immunity (Manz et al., 2005) and mainly consisting of IgG, IgA, and IgM, which can specifically bind antigens and activate complement components (Dunkelberger and Song, 2010). In the sera of untreated patients with PD, the levels of IgA and IgM are significantly decreased compared to those of patients treated with levodopa (Fiszer et al., 1991). Consistent with this, among the peripheral blood mononuclear cells, the amount of those secreting immunoglobulins was also found to be decreased in PD patients (Marttila et al., 1985). Moreover, in parkinsonism-dementia disease among Guamanian populations, serum levels of IgA were higher and those of IgM were lower than those in healthy controls (Hoffman et al., 1981). In addition, increased complement activation has been reported in the SN and serum of PD patients (Bonifati and Kishore, 2007; Orsini et al., 2014).

Non-motor symptoms seriously affect the quality life of PD patients. Among them, fatigue and depressive symptoms might be influenced by inflammatory mechanisms (Lindqvist et al., 2012). At present, studies on the inflammatory biomarkers in the peripheral blood of PD patients mainly focus on proinflammatory cytokines (Gelders et al., 2018). It remains unclear whether immunoglobulins and the complement system contribute to NMSs. In this case-control study, we compared the levels of immunoglobulins (IgG, IgA, and IgM) and complement system components (C3 and C4) between PD patients and healthy controls. We also assessed the relationship between these five indicators and NMSs of patients.

## Materials and Methods

### PD Patients

This study was approved by the Ethics Committee of Qilu Hospital of Shandong University.

Inclusion criteria: patients with Parkinsonian symptoms who met the United Kingdom Brain Bank clinical criteria for PD. Exclusion criteria: (i) patients with a history of regular smoking or alcoholism; (ii) patients with cardiovascular and cerebrovascular disease or hypertension or diabetes complications; (iii) patients with a history of autoimmune disease (involving any of the following systems: thyroid, digestive, kidney, skin, blood and connective tissue); or (iv) patients with acute/chronic infections. In total, 180 sporadic PD patients and 189 healthy controls from the neurology clinic and physical examination center of Qilu Hospital took part in this study with prior and informed consent from 2018 to 2019. According to the Movement Disorders Society (MDS) clinical diagnostic criteria (Postuma et al., 2015), 62 patients were diagnosed with clinically established PD, while 118 patients were diagnosed with clinically probable PD. The Movement Disorder Society-Unified Parkinson’s Disease Rating Scale (MDS-UPDRS)-III and the Non-Motor Symptoms Scale (NMSS) were used to assess patient’s movement disorder and NMSs, respectively. Anti-parkinsonian drugs were administered at a total daily levodopa equivalent dose (LED). To prevent fluctuations in the scores of MDS-UPDRS-III, patients with on/off fluctuations were not included in this study. The basic information and clinical features of the participants are presented in Table 1.

**TABLE 1:** Clinical features of the participants of this study.

**Group**	***n***	**Gender (female/male)**	**Age (year)**	**Duration (year)**	**MDS-UPDRS-III (score)**	**NMSS (score)**	**L-dopa (mg/daily)**
PD patients	180	81/99	61.3 ± 11.9	4.8 ± 3.1	19.7 ± 10.4	12.7 ± 9.1	663.7 ± 460.2
Healthy controls	189	89/100	58.9 ± 14.2	–	–	–	–

*Movement Disorder Society-Unified Parkinson’s Disease Rating Scale (MDS-UPDRS): Non-motor Symptoms Scale (NMSS). Data are expressed as the mean ± standard deviation.*

### Humoral Immunity Indexes

Three milliliters of venous blood was collected in a serum separation hose from the right elbow of each patient between 08:00 am and 10:00 am. After centrifugation, the contents of serum IgG (Ref: 7.00–16.00 g/L), IgM (Ref: 0.40–2.30 g/L), IgA (Ref: 0.70–4.00 g/L), C3 (Ref: 0.80–1.20 g/L), and C4 (Ref: 0.10–0.40 g/L) were detected through the scatter immune turbidity method using the Siemens BN II immunoassay system.

### Statistical Analysis

The levels of serum IgG, IgA, IgM, C3, and C4 were compared between PD patients and healthy controls with the Mann–Whitney test and stratification analysis. The levels of IgG, IgA, IgM, C3, and C4 in patients with different clinical features, including gender (female/male), onset age (≤45 years/>45 years) (Rana et al., 2012), MDS-UPDRS-III scores (≤32/>33) (Martínez-Martín et al., 2015), diagnostic certainty (clinically established/probable PD), and LED (<600/≥600 mg/day) (Brooks, 2008), with or without symptoms in the nine domains of the NMSS, were compared with the Mann–Whitney test. Data are expressed as the median (interquartile range). SPSS 12.0 software (Chicago, IL, United States) was used for the statistical analyses. Bonferroni-adjusted *p* values (Pc) were used to avoid alpha inflation, and Pc < 0.01 and Pc < 0.004 were regarded as statistically significant. Multiple regression models were used to assess the association between indicators of humoral immunity and the clinical features of PD patients. *p* < 0.05 was regarded as statistically significant.

## Results

### The Levels of IgG, IgA, IgM, C3, and C4 Were Similar Between PD Patients and Healthy Controls

The average serum levels of IgG, IgA, IgM, C3, and C4 in patients with PD and healthy controls were all in the normal range. According to the Mann–Whitney test, no significant difference was found among the five indicators between PD patients and healthy controls (*p* > 0.05) (Supplementary Table 1).

### Gender Affects the Levels of IgM, C3, and C4 in PD Patients

**FIGURE 1:**
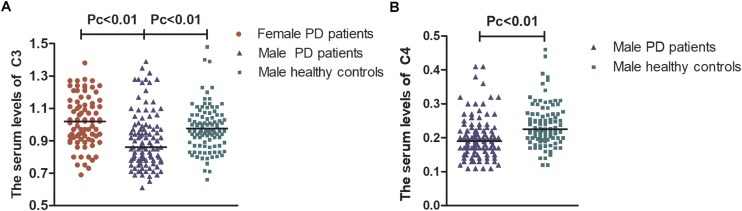
The serum levels of C3 and C4 in female and male PD patients. Male PD patients still had lower levels of C3 and C4 than healthy controls [0.87 (0.22) vs. 0.96 (0.19); 0.19 (0.06) vs. 0.22 (0.07), respectively, Pc < 0.01] and had lower levels of C3 than female PD patients [0.87 (0.22) vs. 1.02 (0.23), Pc < 0.01]. **(A)** Serum levels of C3 were lower in males than in female PD patients and lower than those in male healthy controls. **(B)** Serum levels of C4 were lower in male PD patients than in male healthy controls. Data are presented as the median in the scatter plot and compared by the non-parametric test. Pc, Bonferroni adjusted *p* values, Pc < 0.01.

After stratification analysis with gender, serum levels of the five indicators were similar between females and males in healthy controls (*p* > 0.05). No obvious differences regarding the levels of IgG and IgA were observed in different subtype groups according to gender, including PD groups (female vs. male), female groups (PD vs. HC), and male groups (PD vs. HC), similar to the level of IgM in PD groups (female vs. male) (*p* > 0.05). Interestingly, female PD patients had remarkably higher levels of C3 and C4 than male PD patients [1.02 (0.23) vs. 0.87 (0.22), *p* = 1.90E-5; 0.22 (0.12) vs. 0.19 (0.06), *p* = 0.034; respectively] and higher levels of C3 than female healthy controls [1.02 (0.23) vs. 0.94 (0.22), *p* = 0.014]. Male PD patients had remarkably lower levels of IgM, C3, and C4 than male healthy controls [0.93 (0.44) vs. 1.14 (0.75), *p* = 0.025; 0.87 (0.22) vs. 0.96 (0.19), *p* = 0.001; 0.19 (0.06) vs. 0.22 (0.07), *p* = 5.89E-6; respectively] (Supplementary Table 1). After Bonferroni correction, male PD patients still had lower levels of C3 and C4 than healthy controls [0.87 (0.22) vs. 0.96 (0.19); 0.19 (0.06) vs. 0.22 (0.07), respectively, Pc < 0.01] and a lower level of C3 than female PD patients [0.87 (0.22) vs. 1.02 (0.23), Pc < 0.01] (Figure 1).

### Clinical Features Affect the Levels of IgA, IgM, C3, and C4 in PD Patients

Apart from gender, other clinical features in PD patients were observed, including onset age, MDS-UPDRS-III scores, diagnostic certainty, LED, and different NMSS domains. The levels of IgG, IgA, IgM, C3, and C4 between PD patients with different onset ages (≤45/>45) or diagnostic certainty (clinically established/probable PD) were similar (*p* > 0.05). Patients with higher MDS-UPDRS-III scores (>33 vs. ≤32) or doses of LED (≥600 vs. <600 mg/day) had higher levels of IgA [2.67 (1.45) vs. 2.35 (1.27), *p* = 0.047; 2.57 (1.17) vs. 2.20 (1.06), *p* = 0.010, respectively], while for the levels of IgG, IgM, C3, and C4, no significant differences were observed (Supplementary Table 2).

The percentages of patients who suffered from problems in the nine domains of the NMSS, including cardiovascular falls, sleep/fatigue, mood/cognition, hallucinations, attention/memory, gastrointestinal tract, urinary, sexual function, and miscellaneous, were 36, 70, 63, 10, 53, 72, 35, 28, and 62%, respectively. Compared to patients without symptoms, patients with sleep/fatigue, mood/cognition, attention/memory, gastrointestinal tract, or urinary problems had a higher level of IgM [1.02 (0.7) vs. 0.91 (0.47), *p* = 0.030]; a lower level of C3 [0.92 (0.24) vs. 0.97 (0.27), *p* = 0.045]; lower levels of IgA [1.92 (1.21) vs. 2.57 (0.76), *p* = 0.003], IgM [0.91 (0.44) vs. 1.08 (0.71), *p* = 0.025], C3 [0.89 (0.24) vs. 0.97 (0.23), *p* = 0.001], and C4 [0.19 (0.07) vs. 0.22 (0.09), *p* = 0.014]; a lower level of C4 [0.20 (0.09) vs. 0.22 (0.08), *p* = 0.035]; and lower levels of IgM [0.91 (0.42) vs. 1.05 (0.65), *p* = 0.009] and C3 [0.92 (0.23) vs. 0.96 (0.26), *p* = 0.021], respectively (Supplementary Table 2).

**FIGURE 2:**
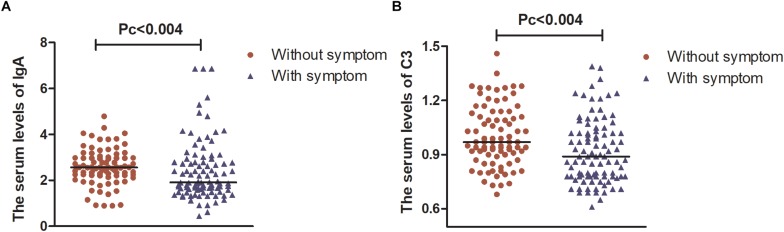
The serum levels of IgA and C3 in patients with PD. **(A)** Serum levels of IgA were lower in patients with attention/memory problems than in those without these problems. **(B)** Serum levels of C3 were lower in patients with attention/memory problems than in those without these problems. Data are presented as the median in the scatter plot and compared by the non-parametric test. Pc, Bonferroni adjusted *p* values, Pc < 0.004.

However, after Bonferroni correction, only patients suffering from attention/memory problems had significantly lower levels of IgA and C3 than those without these problems [1.92 (1.21) vs. 2.57 (0.76); 0.89 (0.24) vs. 0.97 (0.24), Pc < 0.004, respectively, Figure 2].

These results indicated that the humoral immune response of patients with PD was affected by gender, MDS-UPDRS-III scores, LED, and problems in different NMSS domains.

### The Association Between the Five Indicators and the NMSs

To explore the association between the five indicators of humoral immunity and clinical features, multiple regression models involving several factors (gender, age, diagnosis certainty, the course of disease, MDS-UPDRS III score, scores of the nine domains of NMS scale, and LED) were applied in the study. We found that the serum level of IgG was negatively associated with the score of mood/cognition problems (correlation coefficient = −0.209, 95% confidence intervals: −0.408 to −0.010, *p* = 0.039) and was positively associated with the score of gastrointestinal tract problems (correlation coefficient = 0.312, 95% confidence intervals: 0.083 to 0.542, *p* = 0.008) (adjusted *R*^2^ = 0.063, *F* = 1.805, *p* = 0.038). The serum level of C3 was negatively associated with being a male patient (correlation coefficient = −0.100, 95% confidence intervals: −0.159 to −0.041, *p* = 0.001), age (correlation coefficient = −0.003, 95% confidence intervals: −0.006 to 0.000, *p* = 0.007) and the score of sleep/fatigue problems (correlation coefficient = −0.017, 95% confidence intervals: −0.030 to −0.003, *p* = 0.017) (adjusted *R*^2^ = 0.123, *F* = 2.678, *p* = 0.001).

## Discussion

Here, a study involving 369 participants was performed to determine the relationship between serum levels of IgG, IgA, IgM, C3, and C4 and the NMSs of PD. We conducted a case-control study and correlation analyses and found that male PD patients had obviously lower serum C3 levels than male healthy controls and female PD patients. Serum IgG and C3 were correlated with the NMSs of PD.

The incidence of NMSs is high and is associated with a broad range of deficits (Seppi et al., 2019). Sleep disorders and gastrointestinal tract problems are common at the prodromal stage of PD and precede the manifestation of motor symptoms (Gjerstad et al., 2018; Pfeiffer, 2018; Galbiati et al., 2019). Consistent with previous studies, we found that over 50% of patients presented with symptoms in one of the following areas: sleep/fatigue, mood/cognition, attention/memory, gastrointestinal tract, and miscellaneous. Patients with or without NMSs showed different humoral immune responses in terms of serum levels of IgA, IgM, C3, and C4. This implied that humoral immune responses may play a role in the development of NMSs.

Humoral immune responses are influenced by numerous factors. Gaya C. found that the levels of C3 in females were lower than those in males in a healthy Caucasian population (Gaya da Costa et al., 2018). In contrast, Yang X. reported that there was no significant difference in serum C3 levels between females and males in a Chinese Han population (Yang et al., 2018). Similarly, we found no obvious differences in serum C3 levels in healthy controls between genders. However, female patients had higher serum levels of C3 and C4 than male patients. Interestingly, after Bonferroni correction, the serum level of C3 remained higher in female than in male patients. C3 is an important component of the complement system, and its activation is involved in the pathogenesis of PD (McGeer et al., 2017). The absence of C3 in mice did not protect against the depletion of dopaminergic neurons in the toxin-induced MPTP model (Liang et al., 2007). Traditionally, being female is considered a low risk factor for PD (Berg et al., 2015; Picillo et al., 2017). However, females are more likely to suffer from cognitive disorders, pain symptoms, and depression (Georgiev et al., 2017). PD patients who had higher levels of serum C3 and C4 at baseline and maintained consistently high levels over 2 years had a worse quality of life and memory ability (Veselı et al., 2018). After adjusting for other clinical factors, we found that the serum C3 level was inversely correlated with male gender, age, and the severity of sleep/fatigue problems. The levels of C3 were higher in the sleep-deprived human (Hui et al., 2007). Compared to the SLE patients with normal levels of C3, patients with lower C3 had higher FACIT fatigue scale (Li et al., 2017). We speculate that the complement system might be related to the heterogeneity of NMSs in female patients.

IgG autoantibodies of human serum are abundant, with the profiles strongly influenced by age, gender, and specific diseases (Nagele et al., 2013). It has been indicated that IgG is intensely immunolabeled with α-syn in the SN neurons of patients with PD (Orr et al., 2013). No significant difference in the level of serum IgG was found between the healthy controls and PD patients in our study. However, after adjusting for the influence of various confounding factors (gender, age, motor symptoms, NMSs, and doses of levodopa) in the multiple linear regression analysis model, we found that serum IgG levels were negatively associated with mood/cognition problems and were positively associated with gastrointestinal tract dysfunction in PD patients. In patients with generalized anxiety disorder or major depression, the level of serum IgG remained normal (Gold et al., 2012; Islam et al., 2014). Thus, we hypothesized that the pathological changes of PD might contribute to this. In addition, dopamine levels in the putamen and caudate nucleus were negatively correlated with serum IgG in PD patients with constipation (Zhou et al., 2019). This suggests that IgG might be related to NMSs in PD patients, but the mechanism remains complex and unclear. The IgG glycosylation profile of PD patients was different from that of the controls, and the capacity for the IgG to inhibit Fcγ-RIIIa binding might decrease, leading to increased antibody-dependent cell cytotoxicity and a state of low-grade inflammation in PD patients (Russell et al., 2017). Moreover, IgG from patients with PD can induce dopaminergic neuron injury following stereotaxic injection into rat SN (He et al., 2002). However, although intravenous immunoglobulin (IVIG) does not prevent α-synuclein aggregation, it still may reduce α-synuclein neurotoxicity through an unknown mechanism (Smith et al., 2012). Further research is needed to explore the role of serum IgG in the pathogenesis of PD.

Limitations of this study include a short mean course of disease of approximately 5 years and no study reported follow-up on the patients. Further comprehensive studies with longer follow-up durations should be conducted to validate the findings of this study.

## Conclusion

This study profiled the peripheral humoral immune response in patients with PD and revealed that serum C3 and IgG might be correlated with the NMSs of PD.

## Data Availability Statement

The raw data supporting the conclusions of this manuscript will be made available by the authors, without undue reservation, to any qualified researcher.

## Ethics Statement

This study was carried out in accordance with the recommendations of the Ethics Committee of Qilu Hospital of Shandong University with written informed consent from all subjects. All subjects gave written informed consent in accordance with the Declaration of Helsinki. The protocol was approved by the Ethics Committee of Qilu Hospital of Shandong University.

## Author Contributions

CSu, WY, and YmL conceived and designed the research and wrote the manuscript. CSu and WY conducted the experiments. ZZ, CSo, and YnL performed the data collection and statistical analysis. GJ and XW performed data interpretation and the literature search. All authors read and approved the manuscript.

## Conflict of Interest

The authors declare that the research was conducted in the absence of any commercial or financial relationships that could be construed as a potential conflict of interest.
