# Supplementary material for: Peripheral Humoral Immune Response Is Associated With the Non-motor Symptoms of Parkinson’s Disease
Source: Front Neurosci. 2019 Oct 10;13:1057. doi: 10.3389/fnins.2019.01057 (PMC6795918; doi:10.3389/fnins.2019.01057)
Supplement: Supplementary file 1 [file Table_1.DOCX]

Supplement Table 1 The difference of humoral immunity between PD patients and healthy controls

|  | IgG | IgA | IgM | C3 | C4 |
| --- | --- | --- | --- | --- | --- |
| PD Vs. HC | 12.90 (3.98)  12.39 (2.91) | 2.40 (1.22)  2.46 (1.12) | 0.94 (0.55)  1.08 (0.62) | 0.94 (0.28)  0.96 (0.21) | 0.20 (0.08)  0.23 (0.08) |
| HC (female Vs. Male) | 12.50(3.03)  12.02(2.67) | 2.39(1.13)  2.55(1.38) | 1.01(0.66)  1.14(0.75) | 0.94(0.22)  0.96(0.19) | 0.24(0.09)  0.22(0.07) |
| PD (female Vs. male) | 13.30(3.70)  12.80(4.15) | 2.41(1.62)  2.34(1.15) | 0.99(0.81)  0.93(0.44) | *1.02(0.23)  0.87(0.22) | *0.22(0.12)  0.19(0.06) |
| Female (PD Vs. HC) | 13.30(3.70)  12.50(3.03) | 2.41(1.62)  2.39(1.13) | 0.99(0.81)  1.01(0.66) | *1.02(0.23)  0.94(0.22) | 0.22(0.12)  0.24(0.09) |
| Male (PD Vs. HC) | 12.80(4.15)  12.02(2.67) | 2.34(1.15)  2.55(1.38) | *0.93(0.44)  1.14(0.75) | *0.87(0.22)  0.96(0.19) | *0.19(0.06)  0.22(0.07) |

Data are expressed as median (interquartile range). The Mann-Whitney test and stratification analysis are conducted by SPSS 12.0 software. * p<0.05
