# Supplementary material for: Peripheral Humoral Immune Response Is Associated With the Non-motor Symptoms of Parkinson’s Disease
Source: Front Neurosci. 2019 Oct 10;13:1057. doi: 10.3389/fnins.2019.01057 (PMC6795918; doi:10.3389/fnins.2019.01057)
Supplement: Supplementary file 2 [file Table_2.DOCX]

Supplement Table 2 The difference of humoral immunity in PD patients with different clinical features

|  | IgG | IgA | IgM | C3 | C4 |
| --- | --- | --- | --- | --- | --- |
| Gender  (Female/male) | 13.30(3.70)  12.80(4.15) | 2.41(1.62)  2.34(1.15) | 0.99(0.81)  0.93(0.44) | *1.02(0.23)  0.87(0.22) | *0.22(0.12)  0.19(0.06) |
| Onset age  (≤45/＞45) | 12.30(4.31)  13.00(4.10) | 2.51(1.27)  2.40(1.20) | 0.87(0.46)  0.94(0.61) | 0.95(0.31)  0.94(0.27) | 0.21(0.12)  0.20(0.07) |
| MDS-UPDRS-III scores （≤32/＞33） | 13.00(3.9)  12.35(4.89) | *2.35(1.27)  2.67(1.45) | 0.94(0.63)  0.94(0.47) | 0.94(0.28)  0.92(0.25) | 0.2(0.08)  0.19(0.09) |
| Diagnostic certainty (clinically /probable established PD) | 12.3(4.25)  13.2(3.93) | 2.41(1.23)  2.35(1.21) | 0.91(0.71)  0.96(0.51) | 0.94(0.26)  0.94(0.28) | 0.19(0.10)  0.20(0.07) |
| LEDD  (<600/≥600mg/d) | 13.30(4.10)  12.30(3.90) | *2.20(1.06)  2.57(1.17) | 0.94(0.63)  0.94(0.49) | 0.94(0.28)  0.93(0.23) | 0.20(0.11)  0.20(0.06) |
| NMSS part1 symptoms  （without/ with） | 13.00(3.80)  12.8(4.25) | 2.34(1.16)  2.52(1.49) | 0.91(0.56)  1.05(0.65) | 0.95(0.24)  0.92(0.26) | 0.20(0.09)  0.20(0.07) |
| NMSS part2  symptoms  （without/ with） | 13.20(4.05)  12.75(4.00) | 2.24(1.28)  2.4(1.19) | *0.91(0.47)  1.02(0.70) | 0.95(0.35)  0.94(0.22) | 0.20(0.10)  0.20(0.08) |
| NMSS part3  symptoms  （without/ with） | 13.20(3.70)  12.70(3.95) | 2.55(1.24)  2.34(1.21) | 0.91(0.58)  0.98(0.69) | *0.97(0.27)  0.92(0.24) | 0.21(0.09)  0.20(0.08) |
| NMSS part4  symptoms  （without/ with） | 12.85(3.92)  13.45(4.58) | 2.37(1.15)  2.99(2.40) | 0.94(0.57)  1.05(0.59) | 0.94(0.29)  0.96(0.21) | 0.20(0.08)  0.22(0.06) |
| NMSS part5  symptoms  （without/ with） | 12.85(4.10)  12.95(4.57) | *2.57(0.76)  1.92(1.21) | *1.08(0.71)  0.91(0.44) | *0.97(0.24)  0.89(0.24) | *0.22(0.09)  0.19(0.07) |
| NMSS part6  symptoms  （without/ with） | 13.30(4.30)  12.80(4.10) | 2.52(0.93)  2.35(1.39) | 0.93(0.52)  0.94(0.67) | 0.94(0.22)  0.94(0.29) | *0.22(0.08)  0.20(0.09) |
| NMSS part7  symptoms  （without/ with） | 13.2(3.95)  12.20(4.62) | 2.42(1.16)  2.28(1.28) | *1.05(0.65)  0.91(0.43) | *0.96(0.26)  0.92(0.23) | 0.19(0.09)  0.20(0.07) |
| NMSS part8  symptoms  （without/ with） | 13.00(4.00)  12.90(4.10) | 2.34(1.27)  2.41(1.52) | 0.98(0.66)  0.91(0.54) | 0.94(0.27)  0.97(0.29) | 0.20(0.08)  0.20(0.07) |
| NMSS part9  symptoms  （without/ with） | 13.70(3.82)  12.35(3.88) | 2.40(1.06)  2.37(1.32) | 0.94(0.44)  0.98(0.65) | 0.91(0.28)  0.97(0.25) | 0.20(0.07)  0.20(0.09) |

Data are expressed as median (interquartile range). The Mann-Whitney test and stratification analysis are conducted by SPSS 12.0 software. * P<0.05.
